# Family Physicians' Perspectives and Practices on Advance Care Planning in Regional Cities in Japan and the United States: A Convergent Parallel Mixed-Methods Study

**DOI:** 10.7759/cureus.53260

**Published:** 2024-01-30

**Authors:** Keiichiro Kita, Kaku Kuroda, Mayuko Saito, Moe Kuroda, Daishi Ogawa, Maiko Kuroiwa

**Affiliations:** 1 General Internal Medicine, Toyama University Hospital, Toyama, JPN; 2 Family Medicine, State University of New York (SUNY) Upstate Medical University, Syracuse, USA; 3 Public Health, State University of New York (SUNY) Upstate Medical University, Syracuse, USA; 4 Internal Medicine, Nanto Municipal Hospital, Nanto, JPN

**Keywords:** family physician (fp), the united states, japan, convergent parallel mixed method, cross-national research, family medicine, advance care planning

## Abstract

Background: Advance care planning (ACP) has been widely recognized and practiced worldwide since the 1990s. However, only a few studies have compared clinicians' international perceptions of and experiences with ACP. Therefore, this study explored the perceptions and practices of family physicians (FPs) regarding ACP in Japan and the United States.

Methods: We conducted a convergent parallel mixed-methods study using a cross-sectional web-based anonymous questionnaire survey to examine how the perceptions and practices of ACP differ between Japanese and American FPs working in regional cities.

Results: Responses from 20 and 19 FPs in Japan and the United States were obtained, respectively. Both FP groups received ACP training during their residency and practiced ACP with the highest regard for the patient's wishes and values. Quantitative analysis revealed that American FPs placed more emphasis on documentation and patient language skills. Qualitative analysis revealed that Japanese FPs equally emphasized communication with patients' families and with patients. We merged the results of both analyses and hypothesized that the variations in the FPs' approaches to ACP might reflect variations in their backgrounds, such as health insurance systems, cultures, and values in the two countries, rather than differences between individual physicians.

Conclusion: Our study showed that both Japanese and American FPs respect patients' wishes in ACP, with some differences in their perceptions and practices. Therefore, FPs should understand and be flexible with their patients' values and cultural backgrounds as intercultural translators while following appropriate management procedures for successful ACP.

## Introduction

Advance care planning (ACP) has been widely recognized and practiced worldwide since the 1990s [1]. It includes the entire process of discussing future treatment and care extending beyond the preparation of advance directives (ADs). Physicians' attitudes toward end-of-life decisions vary by country [2]. Therefore, understanding the variations in clinical practice in different countries enables us to reflect on our practice and learn different values. However, only a few studies have compared clinicians' international perceptions of and experiences with ACP. Freshman and Yao examined the differences in approaches and perceptions of ACP between multispecialty physicians at a Japanese and an American medical center in 2017 [3]. They revealed that most Japanese physicians and more than half of American physicians did not have an AD on file, although both recognized the importance of ACP. This study aims to explore the perception and practice of ACP in Japan and the United States, focusing on the target population of practicing family physicians (FPs). This research question originated from the experience of one of the authors, who had clinical training in a medium-sized city in the two countries and experienced considerable differences in ACP practices. We conducted a quantitative and qualitative questionnaire survey to clarify the nuanced gaps between these countries. Furthermore, based on the results, we attempted to hypothesize the factors influencing the ACP approach in both countries.

## Materials and methods

Study design

A convergent parallel mixed design was used since it allows for capturing the overall sample trends from the quantitative data and the diversity of the individuals comprising the sample. We conducted a cross-sectional web-based anonymous questionnaire.

Regarding the quantitative analysis, we collected the participants' profiles, clinical backgrounds, and perceptions of ACP through single- or multiple-choice questionnaires adapted or modified from previous literature (Q1-11, 15-23; see Appendices) [2-5]. The agreement of the original question (Q12-14) was measured on a 5-point Likert scale. We used open-ended descriptive questions for the qualitative analysis to collect participants' opinions and experiences regarding ACP (Q24-26).

Although we did not conduct specific assessments, such as test-retest reliability or parallel-form reliability, within the same sample, we assumed that the validated questions developed from previous literature were reliable [2-5]. To verify the face validity of the questionnaire, we reviewed the questions among the co-authors for clarity, use of appropriate language, question order, and variety of options. Subsequently, we conducted a pilot test with 130 members of the Chubu Branch of the Japanese Primary Care Society who attended the online meeting in 2022. Based on the results, we removed questions that asked for opinions on domestic issues and case scenarios and changed misleading language. Additionally, we obtained feedback from American FPs (Dr. JK and Dr. RB; see Acknowledgments) on whether the questions in English would elicit the intended responses. Finally, we re-reviewed the Japanese and English versions and launched the survey (Appendices).

Participant recruitment

This study included FPs from Japan and the United States. FPs are appropriate individuals to investigate the current situation of ACPs since they have a comprehensive relationship with their patients and a rich ACP experience [6]. The respondents were restricted to those who met our criteria to obtain a qualitatively homogeneous sample: they had to be board-certified FPs working in Syracuse, USA, or Toyama, Japan. Syracuse and Toyama are medium-sized regional cities with similar working environments (the metropolitan areas of Syracuse and Toyama have approximately 660,000 and 540,000 people, respectively). The questionnaire survey was initiated in February 2023. We were acquainted with several FPs in Syracuse and Toyama who would be suitable participants for this study. Therefore, we initially asked them to participate in this study and subsequently increased the number of participants by selecting participants who met our criteria from the candidates they referred to us (purposive sampling) [7]. Participants' informed consent to participate was obtained at the top of the anonymous questionnaire. We conducted a sequential thematic analysis concurrently with data sampling [8]. Recruitment was discontinued in April 2023 when we found that similar themes reappeared in the thematic analysis and we could not find any new ones. Since this was an observational study, the sample size was considered sufficient for group-by-group comparisons, and power analyses were not conducted.

Data analysis

We used Fisher's exact test and Wilcoxon rank-sum test for quantitative analysis to compare the two groups, using IBM SPSS Statistics for Windows, Version 28.0 (Released 2021; IBM Corp., Armonk, New York, United States). Statistical significance was set at p<0.05 with a two-tailed test.

A thematic analysis [8] was conducted for the qualitative analysis based on the phenomenological paradigm as a theoretical framework. All authors were FPs or general practitioners with experience in qualitative research and ACP. Four of the authors used MAXQDA 2022® (VERBI Software, 2021), a software program designed for computer-assisted qualitative and mixed-methods data analysis. Each author coded and stratified the text data separately to identify common themes and patterns among the respondents. After analysis, several meetings were held to revise and integrate the codes with the themes. The Ethical Review Boards of Toyama University Hospital (approval number: R2022106) and State University of New York (SUNY) Upstate Medical University (approval number: 1956963-1) approved this study's protocol.

## Results

We received responses from 20 and 19 FPs in Japan and the United States, respectively.

Quantitative data analysis

Table 1 presents the respondents' profiles (Q1-11). No statistical differences were found in age, sex, clinical experience, ACP education, and ACP experience, between the two groups. Contrarily, their ethnicity, religion, clinical setting, and incentives for ACP were statistically different. 

**Table 1:** Characteristics of respondent FPs Fisher's exact test and Wilcoxon rank-sum test were used for quantitative analysis to compare the two groups. Statistical significance was set at p<0.05 (*) with a two-tailed test. Years since post graduation are expressed as mean±standard deviation and others as numbers (n) and percentages (%) n: number; ACP: advance care planning; PGY: postgraduate year: ns: not significant: FPs: family physicians

Participants' characteristics	Category	Japan (n=20)	United States (n=19)	p-value
Years since post graduation (mean±SD)		15.5±7.7	23.4±13.6	0.17
Years since post graduation (PGY) category n (%)	PGY 1-10	8 (40)	4 (21.1)	0.30
	PGY >10	12 (60)	15 (78.9)	0.30
Sex n (%)	Male	14 (70)	10 (52.6)	0.33
	Female	6 (30)	9 (47.4)	0.33
Ethnicity n (%)	European	0 (0)	12 (63.2)	<0.01*
	Asian	20 (100)	3 (15.8)	<0.01*
	Latino	0 (0)	2 (10.5)	0.23
	Others	0 (0)	2 (10.5)	0.23
Religion n (%)	Catholic	0 (0)	5 (26.3)	0.02*
	Christian	1 (5)	3 (15.8)	0.34
	Buddhism	7 (35)	0 (0)	<0.01*
	Shinto	1 (5)	0 (0)	1.00
	None	11 (5)	4 (21.1)	0.048*
	Others	0 (0)	7 (36.6)	<0.01*
Primary practice setting n (%)	Outpatient	6 (30)	18 (94.7)	<0.01*
	Inpatient	4 (20)	0 (0)	0.11
	House call	8 (40)	0 (0)	<0.01*
	Emergency room	1 (5)	0 (0)	1.00
	Outpatient/inpatient	1 (5)	1 (5.3)	1.00
Training or education on ACP n (%)	Yes, as a medical student	4 (20)	7 (36.6)	0.30
	Yes, as a resident	18 (90)	18 (94.7)	1.00
Incentives (bill for ACP visit) n (%)	Yes, there is	4 (20)	11 (57.9)	0.02*
	No, there is none	13 (65)	1 (5.3)	<0.01*
Frequency of ACP discussion n (%)	Almost every day	4 (20)	3 (15.8)	1.00
	Several times weekly	3 (15)	3 (15.8)	1.00
	Several times monthly	6 (30)	7 (36.8)	0.74
	Several times yearly	7 (35)	6 (31.6)	0.74
	Not at all (no experience)	0 (0)	0 (0)	1.00

**Figure 1:**
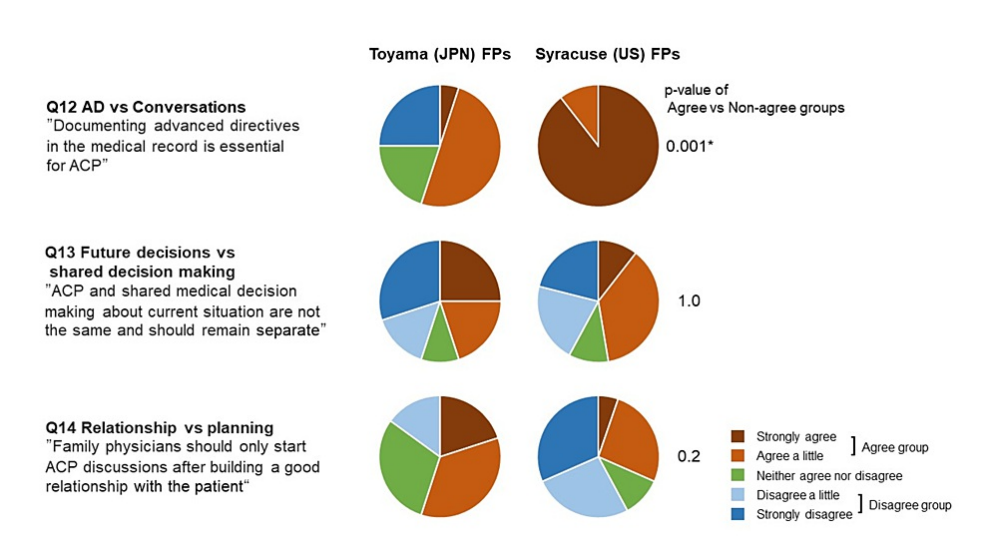
Perceptions of FPs in both countries on the conflict items and our original question Conflict items were those items in which the Delphi panel did not reach a consensus. P-values are the results of Fisher's exact and Wilcoxon rank-sum tests for the "agree" vs. "disagree" groups. Statistical significance was set at p<0.05 (*) with a two-tailed test AD: advance directives; ACP: advance care planning; FP: family physicians; JPN: Japan; US: United States

Figure 1 shows the perceptions of FPs in the two countries on items where the Delphi panel did not reach a consensus [9] (Q12 and 13) and our original question (Q14). American FPs placed more emphasis on documenting ADs than Japanese FPs (p<0.05). Both groups had similar percentages of agreement with the idea that ACP differs from current shared decision-making. In an original question regarding "Relationships vs. Planning," the Japanese FPs were more likely to respond that ACP discussions should begin after good relationships have been established than American FPs, although not statistically significant in the "agree" vs. "non-agree" cross-tabulation.

**Figure 2:**
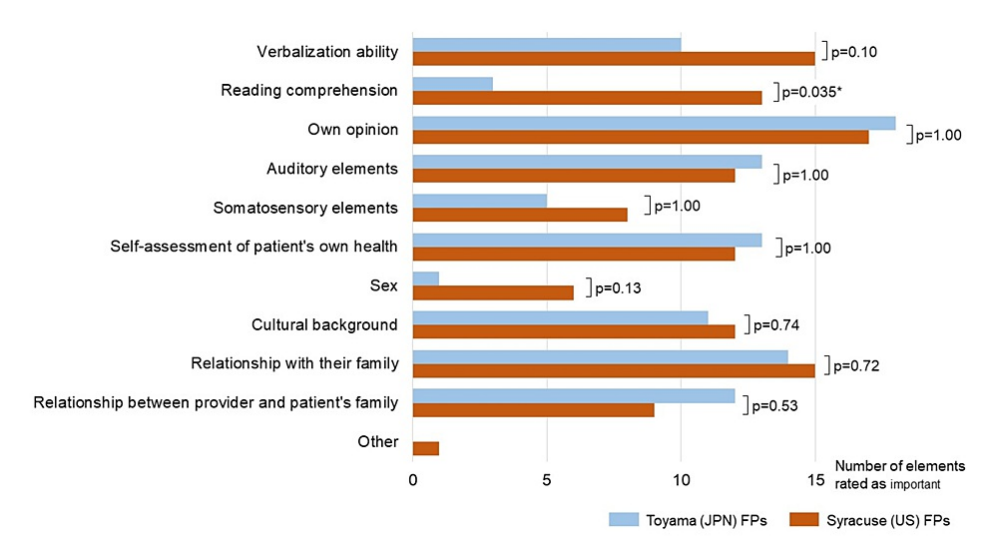
Elements of patient communication that FPs consider important in implementing Fisher's exact test and Wilcoxon rank-sum test were used for quantitative analysis to compare the two groups. Statistical significance was set at p<0.05 (*) with a two-tailed test JPN: Japan; US: United States; FP: family physician

Figure 2 shows the communication elements identified as important by the FPs in implementing ACP (Q15). American FPs considered the patient's ability to understand textual information more critical than the Japanese FPs (p=0.035). Regarding facilitators or barriers to ACP discussions, 85% and 52% of the Japanese and American FPs responded that the family/key person's opinion could influence ACP discussions (Q20-22, p=0.04). Responses to the remaining questions (Q16-23) were not statistically different between the two groups (data not shown).

Qualitative data analysis

Table 2 shows the thematic analysis results regarding the true outcomes and evaluation of ACP, best experience, and greatest challenge in practicing ACP (Q24-26).

**Table 2:** Results of thematic analysis n: number; ACP: advance care planning; MOLST: Medical Orders for Life-Sustaining Treatment

Question	Country	Theme	n	Subcategory
What are the true outcomes of ACP, and how should it be evaluated?	JPN	Patient's satisfaction	10	End-of-life acceptance
		Family's satisfaction	6	Family's sense of ease and reduced burden
		Physician's satisfaction	2	Reflection
	USA	Patient's wishes	9	Patient's values and autonomy
		Shared understanding	4	Frequent discussion
		Documentation	2	MOLST form
What is the best experience of practicing ACP?	JPN	Fulfilled patient's wishes	8	Dying at home
		Comfortable handling	7	Handover and preparedness for bereavement
		Family's satisfaction	4	Peace of mind and gratitude
		Physician's reflection	3	Knowledge of life
	USA	Fulfilled patient's wishes	11	Autonomy and empowered self-decision making
		Understand the patient's value of life	7	In-depth conversation and preparation
		Obtaining documentation	2	MOLST form and written wishes
What was your biggest challenge in practicing ACP?	JPN	Unnatural communication	5	Puzzlement, embarrassment, and uncomfortableness
		Negative reaction from the patient's family	5	Anger, refusal, claim, and conflict
	USA	Lack of time	8	Hard to achieve a nuanced understanding
		Negative reaction from the patient's family	7	Defensiveness and refusal

Japanese FPs listed patient satisfaction first as a true outcome of ACP, followed by family and physician satisfaction. In contrast, American FPs listed patient wishes first, followed by mutual understanding and documentation, such as the Medical Orders for Life-Sustaining Treatment (MOLST) form. None of the groups commented on specific evaluation methods. The two groups mentioned fulfilling the patient's wishes as what FPs considered their best ACP experience. Japanese FPs also stated that ACP provided a well-structured framework for dealing with stressful situations, such as handovers and acute exacerbations. As for the most significant challenge in practicing ACP, Japanese FPs indicated that it changed their communication with patients and families into an unnatural and awkward form. On the other hand, American FPs stated that they could not spend enough time on ACP. Both groups mentioned that ACP sometimes leads to negative reactions from patients and their families. The following are the extracted themes and quotations from the responses to Q25 and 26:

Fulfilled Patient's Wishes

"We supported the patient's wish to stay at home until the end of his life, enjoying his favorite pastimes together with his family, such as smoking and talking with his family." (25 years of experience (YOE), Japanese female)

"Patient in hospital with terminal cancer, still able to move, asked to be removed from life support and goes home to die with family and hospice care." (45 YOE, Christian European male)

"A patient feeling empowered to make decisions regarding their health care and to communicate that with their loved ones and healthcare team." (13 YOE, Catholic European female)

Comfortable Handling

"The handover went well. We can treat each other with the same values even if there is a change of doctors." (17 YOE, Buddhist Japanese male)

Physician's Reflection

"It led to grief counseling for the family as well as good treatment experience for the medical staff involved." (9 YOE, Japanese female)

Family Satisfaction

"Family members who supported the patient expressed satisfaction that they could be with the patient until death." (35 YOE, Japanese male)

Understand the Patient's Value of Life

"A patient whom I know well and who is open to discussion without strong preconceptions, who is willing to make decisions, and who I am confident understands what is being decided." (32 YOE, Christian male)

Obtaining Documentation

"Getting MOLST with a patient who was repeatedly admitted. Eventually, she elected to be Do Not Resuscitate/Do Not Intubate and remained comfortable at her residence." (26 YOE, Asian female)

Unnatural Communication

"When I talked about the patient's prognosis and future before we had established a relationship, the family members looked at me suspiciously or said, 'This is too sudden for us'." (9 YOE, Japanese female)

Negative Reactions from the Patient's Family

"The family's anger was temporarily directed at the attending physician during a conference involving the family." (16 YOE, Buddhist Japanese male)

"When a family disagree with each other or provide shaming to the patient." (25 YOE, European Christian male)

Lack of Time

"Lack of time, it difficult to effectively discuss ACP at their annual appointments since other preventive care discussions were ongoing." (14 YOE, Asian Islamic female)

## Discussion

This study combined hypothesis testing and theme extraction in a single study by employing the convergent parallel mixed research method. Quantitative analysis showed no statistically significant differences between sampled Japanese (Toyama) and American (Syracuse) FPs in education or practice opportunities for ACP. The clinical environment of FPs in Japan was more diverse than in the United States. Japanese FPs provide inpatient care, outpatient care, and home visits in rural hospitals. In the scale questionnaire, American FPs emphasized AD and the patient's reading comprehension ability (statistically significant). Additionally, American FPs tended to focus on the patient's sex (not statistically significant); however, the multiple-choice questionnaire could not reveal the reason. Japanese FPs emphasized dialogue and relationships with the patient, but no significant differences existed. Qualitative analysis identified 12 themes concerning the perspectives and practices of ACP. FPs in both countries respected patients' wishes. American FPs emphasized patient autonomy, while Japanese FPs emphasized patient and family satisfaction.

We synthesized the results of both qualitative and quantitative studies and conducted a meta-inference [10] using the literature (Table 3). We hypothesized that the differences in the FPs' approaches to ACP in the two countries might reflect variations in each country's background, such as health insurance systems, cultures, and values, rather than differences between individual physicians. The text in quotation marks below refers to the themes and subcategories related to the meta-inference.

**Table 3:** Joint display of merged results and meta-inference Meta-inference diagram integrating qualitative and quantitative data with hypotheses. The italics in the quotation are themes extracted by thematic analysis. Fisher's exact test and Wilcoxon rank-sum test were used for quantitative analysis to compare the two groups. Statistical significance was set at p<0.05 (*) with a two-tailed test n: number; ACP: advance care planning; FP: family physician; AD: advance directives

Meta-inference and data		Toyama (Japan) FPs			Syracuse (the United States) FPs		
Approach to ACP inferred from the merged data		Patient and family satisfaction-focused approach: Japanese FPs emphasize communication with patients as members of their family unit in a high-context culture. They routinely implement ACP to fulfill patients' wishes, but the business-like approach leads to uncomfortable relationships. Japanese FPs believe that patient and family acceptance of the current situation and staying at home lead to their satisfaction.			Patient autonomy and document-based approach: American FPs emphasize patient autonomy and shared understanding with documentation in a low-context culture. They routinely implement ACP to fulfill the patient's wishes, but they are frustrated by the limited time for discussion in health insurance systems. American FPs believe empowering patients to make self-decisions leads to their satisfaction.		
Quantitative data: questions and responses	Questions	Answered yes or agree n (%)					p-value
	Training on ACP as a resident (yes/no)	18 (94.7)			18 (90)		1.00
	ACP discussion: daily to several times per month (yes/no)	13 (65)			13 (68.4)		1.00
	No experience of ACP (yes/no)	0 (0)			0 (0)		1.00
	AD is essential for ACP (agree/disagree)	11/20 (55)			19/19 (100)		<0.01*
	Reading comprehension of a patient is important (agree/disagree)	3/20 (15)			13/19 (68.4)		<0.01*
Qualitative data: questions and themes extracted by thematic analysis	Questions	Theme	n		Theme	n	
	What are the true outcomes of ACP?	"Patient's satisfaction"	10		"Patients' wishes"	9	
		"Family's satisfaction"	6		"Shared understanding"	4	
		"Physician's satisfaction"	2		"Documentation"	2	
	What is the best experience of practicing ACP?	"Fulfilled patient wishes"	8		"Fulfilled patient wishes"	11	
		"Comfortable handling"	7		"Understand the patient's value of life"	7	
		"Family's satisfaction"	4		"Obtaining documentation"	4	
		"Physician's reflection"	3		None	0	
	What was your biggest challenge in practicing ACP?	"Unnatural communication"	5		"Lack of time"	5	
		"Negative reaction from the patient's family"	5		"Negative reaction from the patient's family"	5	
Background factors assumed to influence ACP practice and association with the data	Factors	Characteristic and associated data					
	Financial incentives for ACP	Absent: vague limitations on interview time			Exist: clear limitations on interview time		
	Communication culture	High context: "unnatural communication"			Low context: "documentation" and reading ability		
	Valance of value	Family oriented: "family's satisfaction"			Individual oriented: "patient autonomy"		

Regarding health insurance, Japan has a national health insurance system with reasonable premiums that cover all citizens. However, the reimbursement of physician time for each patient remains unclear, and ACP performance has no direct reward as of 2023. ACP is yet to be widely practiced under this system in Japan [11]. The American insurance system (both private and Medicare) provides financial incentives for FPs to engage in ACP [12]. These payment systems encourage physicians to care for more patients but spend less time with them [13] (referred to as "lack of time").

Communication cultures in Japan and the United States differ on several scales [14]. These variations may originate from cultural differences between Asian and Western communication approaches. Japan, almost a mono-ethnic nation with a long-shared history, has one of the world's highest-context cultures [14,15]. High-context culture is a relational society that requires reading between the lines since messages are nuanced, layered, and implied rather than stated [14]. It is, therefore, very important to be able to read the person's emotions in high-context culture. In this survey, Japanese (Toyama) FPs heavily weighted "patient's satisfaction" and "family's satisfaction" when they implemented ACP. Although having an AD is useful in the handover (referred to as "comfortable handling"), a businesslike approach leads to "unnatural communication" in Japan. However, the United States, a multi-ethnic rational nation with a short history, has the world's lowest-context culture [14]. A low-context culture is a transparent, verbal-oriented society where messages are taken at face value. American (Syracuse) FPs more heavily weighted "obtaining documentation," "shared understanding," and patient's reading comprehension in ACP.

According to the World Values Survey [16], Japanese and American citizens value their families. However, the Japanese focus on the harmonious functioning of the family ("family's satisfaction"), whereas Americans are more concerned about individual autonomy [17] ("autonomy and empowered self-decision making," "understanding the patient's value of life"). Japanese, similar to other Asians or Latinos, consider a patient's illness as an issue for the entire family [18,19]. In this collective paradigm, decisions are made in the context of family and surrounding relationships rather than individual autonomy [20,21]. Additionally, fear of conflict with families could be a barrier to the spread of ACP in Asian countries [22-24]. Given the above, the perception of "negative reactions from the patient's family" may differ between Japanese and American FPs. However, we could not determine this in the present study.

Although many discussions have been conducted [25], various studies have shown that ACP positively impacts the quality of end-of-life care [26-28]. Our study supports the importance of qualitative measures in measuring ACP outcomes, such as patient autonomy and patients' and families' subjective satisfaction. Additionally, the ACP experience has positively impacted the lives of some Japanese physicians (physician's reflection), which may be an internal incentive for physicians to implement ACP. Furthermore, Japanese FPs can improve ACP quality by encouraging documentation, such as ADs, while respecting Japanese cultural values and norms [29,30].

Limitations

This study's participants were recruited within the regional city area in each country using purposive sampling. Therefore, the results derived from this small sample-sized study should be cautiously interpreted to avoid overgeneralization. Moreover, further studies are warranted to implement ACPs that are appropriate for each country's sociocultural context.

## Conclusions

Both FPs in Japan (Toyama) and the United States (Syracuse) implemented ACP with respect for patients' wishes, although some differences existed in their approaches to ACP. As intercultural translators in a rapidly globalizing society, FPs should understand and be flexible with their patients' values and cultural backgrounds. Therefore, we believe that understanding the differences in the clinical practice of ACP between Japan and the United States can enable us to self-reflect, recognize each other, and contribute to improving healthcare quality.

## Appendices

Questionnaire about advance care planning (ACP) for family physicians

Dimension 1: Profile

1.   Year of medical school graduation

2.   Sex

3.   Ethnicity

1)       Asian

2)       European

3)       African

4)       Latino

5)       Others

4.   Religion

1)       Buddhism

2)       Shinto

3)       Catholic

4)       Christian

5)       Jewish

6)       Islam

7)       Others

8)       None/NA

Dimension 2: Clinical Background

5.   Primary practice setting

1)       House call (home visit care)

2)       Outpatient clinic

3)       Hospital (inpatient care)

4)       ER

5)       Others

6.   How frequently do you discuss ACP?

1)       Not at all

2)       Several times yearly

3)       Several times monthly

4)       Several times weekly

5)       Almost every day

Dimension 3: The ACP Training

7.   Did you receive any training or education on ACP as a medical student?

1)       Yes, I did

2)       No, I did not

3)       I do not know

8.   Did you receive any training or education on ACP during residency?

1)       Yes, I have

2)       No, I have not

3)       I do not know

9.   Are you aware of any incentives for discussing ACP (do you bill for the ACP visit)?

1)       Yes, there is

2)       No, there is none

3)       I do not know

10.   Do you engage in self-directed learning regarding ACP (literature review and conference participation, among others)?

1)       Yes, always

2)       Yes, sometimes

3)       No, never

11.   Do you use dedicated tools or programs (such as Serious Illness Conversation Guide (SICG)) when conducting ACP?

1)       Yes

2)       No

3)       I do not know

Dimension 4: Perceptions of ACP

Please select the most applicable answer.

12.   "Advance directive vs. conversations": Documenting advance directives in the medical record is essential for ACP.

1)       Strongly agree (Documentation is more important than conversations)

2)       Agree a little

3)       Neither agree nor disagree

4)       Disagree a little

5)       Strongly disagree (Conversations are more important than documentation)

13.   "Future decisions vs. shared decision-making": ACP and shared medical decision-making about the current situation are not the same and should remain separated.

1)       Strongly agree (Real-time decision-making and the ACP are different)

2)       Agree a little

3)       Neither agree nor disagree

4)       Disagree a little

5)       Strongly disagree (Real-time decision-making is a part of the ACP continuum)

14.   "Relationship and advance care planning": Family physicians should only initiate ACP discussions after building a good relationship with patients.

1)       Strongly agree (Good relationships first, followed by ACP)

2)       Agree a little

3)       Neither agree nor disagree

4)       Disagree a little

5)       Strongly disagree (ACP can promote good relationships)

Factors affecting ACP:

15.    What elements of patient communication are you focusing on when implementing ACP? Please select all that apply.

1)       Patient's comprehension of verbal discussions

2)       Patient's comprehension of written information

3)       Patient's opinion (the words that actually come out of the patient)

4)       Auditory elements (tone of voice and the way of speaking)/visual elements (facial expression and eye contact)

5)       Somatosensory elements (posture and gestures)

6)       Self-assessment of patient's health

7)       Patient's sex

8)       Patient's cultural background

9)       Relationship between patient and their family

10)     Relationship between provider and patient's family

11)      Others

16.    Considering the patient's background, what promotes ACP discussions? Please select all that apply.

1)        Diagnosis of patient's disease

2)       Age

3)       Patient's self-understanding of their disease

4)        Religion and faith

5)        Financial situation

6)        Education level

7)        Others/none of the above

17.     If you selected "others" for the above question, please write your comments or opinions here.

18.    Considering the patient's background, what are some barriers to ACP discussions? Please select all that apply.

1)        Diagnosis of patient's disease

2)        Age

3)        Patient's self-understanding of their disease

4)        Religion and faith

5)        Financial situation

6)        Education level

7)        Others/none of the above

19.    If you selected "others" for the above question, please write your comments or opinions here.

20.    Other than the patient's background, what promotes ACP? Please select all that apply.

1)        Family/key person's opinion

2)        Provider-patient relation

3)        Expected prognosis

4)        Healthcare insurance system

5)        Zeitgeist/the spirit of the ages

6)         Others

21.    If you selected "others" for the above question, please write your comments or opinions here.

22.    Other than the patient's background, what inhibits ACP (barriers of ACP)? Please select all that apply.

1)        Family/key person's opinion

2)        Provider-patient relation

3)        Expected prognosis

4)        Healthcare insurance system

5)        Zeitgeist/the spirit of the ages

6)         Others

23.    If you selected "others" for the above question, please write your comments or opinions here.

Dimension 5: Opinions and Experience on ACP

24.    What are the true outcomes of ACP, and how should they be evaluated?

25.    What is the best experience of practicing ACP? Please briefly describe one episode below.

26.    What was your biggest challenge in practicing ACP? Please briefly describe one episode below.

## References

[1] Emanuel LL, Danis M, Pearlman RA, Singer PA (1995). Advance care planning as a process: structuring the discussions in practice. J Am Geriatr Soc.

[2] Miccinesi G, Fischer S, Paci E (2005). Physicians' attitudes towards end-of-life decisions: a comparison between seven countries. Soc Sci Med.

[3] Freshman B, Yao A (2017). Assessing the knowledge, perceptions and practices in advance care planning: a comparison between American and Japanese physicians. Hos Pal Med Int Jnl.

[4] Howard M, Day AG, Bernard C, Tan A, You J, Klein D, Heyland DK (2018). Development and psychometric properties of a survey to assess barriers to implementing advance care planning in primary care. J Pain Symptom Manage.

[5] Malhotra C, Chaudhry I (2023). Barriers to advance care planning among patients with advanced serious illnesses: a national survey of health-care professionals in Singapore. Palliat Support Care.

[6] Ji TA, Ho J, McGregor MJ, Kow J (2020). Family physicians' perspectives on advance care planning in community-dwelling elderly patients. Can Fam Physician.

[7] Campbell S, Greenwood M, Prior S (2020). Purposive sampling: complex or simple? Research case examples. J Res Nurs.

[8] Alhojailan MI (2012). Thematic analysis: a critical review of its process and evaluation. W East J Soc Sci.

[9] Sudore RL, Lum HD, You JJ (2017). Defining advance care planning for adults: a consensus definition from a multidisciplinary Delphi panel. J Pain Symptom Manage.

[10] Fetters MD, Molina-Azorín JF (2019). A checklist of mixed methods elements in a submission for advancing the methodology of mixed methods research. J Mix Methods Res.

[11] Nakayama T, Yoshida T, Mori M (2021). The physicians' barriers to practice of advance care planning: a single facility questionnaire survey. Palliat Care Res.

[12] Luth EA, Manful A, Weissman JS, Reich A, Ladin K, Semco R, Ganguli I (2022). Practice billing for Medicare advance care planning across the USA. J Gen Intern Med.

[13] Aydin S (2019). The effects of ownership structure on time spend by physicians with patients. Eur J Environ Public Health.

[14] Meyer E (2014). The culture map: breaking through the invisible boundaries of global business. Affairs.

[15] Kim D, Pan Y, Park HS (1998). High-versus low-context culture: a comparison of Chinese, Korean, and American cultures. Psychol Mark.

[16] Haerpfer C, Inglehart R, Moreno A (2023). World Values Survey Wave 7 (2017-2022). Austria: JD Systems Institute & WVSA Secretariat.

[17] Matsumura S, Bito S, Liu H, Kahn K, Fukuhara S, Kagawa-Singer M, Wenger N (2002). Acculturation of attitudes toward end-of-life care: a cross-cultural survey of Japanese Americans and Japanese. J Gen Intern Med.

[18] Candib LM (2002). Truth telling and advance planning at the end of life: problems with autonomy in a multicultural world. Fam Syst Health.

[19] Blackhall LJ, Murphy ST, Frank G, Michel V, Azen S (1995). Ethnicity and attitudes toward patient autonomy. JAMA.

[20] Turoldo F (2010). Relational autonomy and multiculturalism. Camb Q Healthc Ethics.

[21] Morita T, Oyama Y, Cheng SY (2015). Palliative care physicians' attitudes toward patient autonomy and a good death in East Asian countries. J Pain Symptom Manage.

[22] Gómez-Vírseda C, de Maeseneer Y, Gastmans C (2019). Relational autonomy: what does it mean and how is it used in end-of-life care? A systematic review of argument-based ethics literature. BMC Med Ethics.

[23] Abe A, Kobayashi M, Kohno T, Takeuchi M, Hashiguchi S, Mimura M, Fujisawa D (2021). Patient participation and associated factors in the discussions on do-not-attempt-resuscitation and end-of-life disclosure: a retrospective chart review study. BMC Palliat Care.

[24] Martina D, Lin CP, Kristanti MS (2021). Advance care planning in Asia: a systematic narrative review of healthcare professionals' knowledge, attitude, and experience. J Am Med Dir Assoc.

[25] Morrison RS, Meier DE, Arnold RM (2021). What's wrong with advance care planning?. JAMA.

[26] Detering KM, Hancock AD, Reade MC, Silvester W (2010). The impact of advance care planning on end of life care in elderly patients: randomised controlled trial. BMJ.

[27] Brinkman-Stoppelenburg A, Rietjens JA, van der Heide A (2014). The effects of advance care planning on end-of-life care: a systematic review. Palliat Med.

[28] Martin RS, Hayes B, Gregorevic K, Lim WK (2016). The effects of advance care planning interventions on nursing home residents: a systematic review. J Am Med Dir Assoc.

[29] Miyashita J, Shimizu S, Shiraishi R (2022). Culturally adapted consensus definition and action guideline: Japan's advance care planning. J Pain Symptom Manage.

[30] Chikada A, Takenouchi S, Nin K, Mori M (2021). Definition and recommended cultural considerations for advance care planning in Japan: a systematic review. Asia Pac J Oncol Nurs.

